# Mid-term outcomes of an alternative remodelling technique for aortic root replacement without coronary ostial mobilisation or reimplantation

**DOI:** 10.1186/s13019-022-02051-x

**Published:** 2023-02-01

**Authors:** Leonidas Hadjinikolaou, Metesh Acharya, Carmelo Dominici, Fausto Biancari, Furqan Raheel, Aamer Ahmed, Giovanni Mariscalco

**Affiliations:** 1 Department of Cardiac Surgery, Glenfield Hospital, Groby Road, Leicester, LE3 9QP UK; 2 Department of Cardiovascular Surgery, Campus Bio-Medico University of Rome, Via Álvaro del Portillo, 21, 00128 Rome, Italy; 3 Heart and Lung Center, Helsinki University Hospital and University of Helsinki, Haartmaninkatu 4, P.O. Box 340, 00029 Helsinki, Finland; 4 Department of Anaesthesia, Glenfield Hospital, Groby Road, Leicester, LE3 9QP UK

**Keywords:** Aortic root remodelling, Aortic root replacement, Bentall procedure

## Abstract

**Background:**

We compare the early and late outcomes of a modified aortic root remodelling (ARR) technique for aortic root replacement without mobilisation or reimplantation of the coronary ostia, with those of the modified Bentall-de Bono procedure.

**Methods:**

A retrospective observational study was performed comprising 181 consecutive patients who underwent aortic root replacement with a modified Bentall-de Bono procedure (104 patients) or ARR (77 patients) between January 2013 and December 2019. Primary endpoints included hospital mortality and late survival. Secondary endpoints included incidence of post-operative complications and freedom from late re-operation.

**Results:**

ARR procedures were performed with shorter cross-clamp times and comparable cardiopulmonary bypass times to modified Bentall-de Bono procedures. The incidence of early post-complications was comparable between groups. 30-day mortality was numerically lower with ARR than the modified Bentall-de Bono procedure. Over 7-year follow-up, 4 patients (3.8%) required repeat aortic surgery after a modified Bentall-de Bono procedure, and none after ARR. Long-term mortality after ARR and after modified Bentall-de Bono procedures was 17.1% and 22.7%, respectively. The cumulative incidence of reintervention on the aortic root/valve was 3.2% after a modified Bentall-de Bono procedure and 0% after ARR. When adjusted for other independent risk factors, late mortality was not influenced by the procedure performed, although competing risk adjusted for age showed that the modified Bentall-de Bono procedure was associated with an increased risk of aortic root/aortic valve re-operation.

**Conclusions:**

The modified ARR technique is associated with reduced myocardial ischaemia time, lower post-operative mortality and aortic re-intervention rates compared to a modified Bentall-de Bono procedure. It may be considered a safe and feasible procedure for aortic root/ascending aortic replacement offering good long-term outcomes.

## Background

Aortic root replacement according to the modified Bentall-de Bono procedure is considered the standard surgical technique for disease affecting the aortic valve and sinuses of Valsalva simultaneously [1–4]. Although the modified Bentall-de Bono procedure is usually associated with excellent early outcomes, higher mortality and morbidity rates can be expected in elderly populations with multiple comorbidities [1, 5–7]. In addition, false aneurysm formation and progression of aneurysmal disease are predominant causes for late re-operations after aortic root replacement [8–12]. Our group recently introduced a new technique for the replacement of the ascending aorta and aortic root remodelling (ARR) without coronary artery mobilisation or reimplantation, to reduce operative time and the risk of peri-operative bleeding [13].

In the present study, we compared the early and late results our root remodelling technique in patients affected by concomitant diseases of the aortic valve and sinuses of Valsalva, with those of the modified Bentall-de Bono procedure.

## Methods

### Patient population

We performed a retrospective observational study comprising all patients aged ≥ 18 years affected by sinus of Valsalva dilatation and disease of the aortic valve not amenable to repair from January 2013 to December 2019. Exclusion criteria encompassed patients affected by acute type A aortic dissection and those with an open or hybrid repair of the descending thoracic aorta. Indications for aortic surgery were in accordance with the European guidelines for the diagnosis and treatment of aortic diseases [1]. For each patient, baseline characteristics, demographics, comorbidities, intra-operative factors, early and late post-operative outcomes data were collected. Aortic measurements were obtained by pre-operative computed tomography (CT) imaging in all patients and included diameter at the level of sinuses of Valsalva, the sino-tubular junction and mid-ascending aorta [1].

Data were prospectively collected, validated, and stored by a data management team at our institution, as part of the UK National Institute for Cardiovascular Outcomes Research (NICOR) Registry. Late survival data after discharge were obtained from the UK Office of National Statistics. The study protocol was in compliance with the local Institutional Clinical Audit Review Board (Ref No 11276) and patient consent was waived.

### Outcome measures and definitions

Primary endpoints included in-hospital mortality and late survival. Main secondary endpoints included re-operation for bleeding/tamponade, post-operative neurologic and renal complications, sternal wound infection, length of hospital stay, and freedom from late re-operation.

Variables were defined according to the European System for Cardiac Operative Risk Evaluation II definition criteria and outcome endpoints according to current Valve Academic Research Consortium-2 (VARC-2) definitions and guidelines for reporting mortality and morbidity after cardiac valve interventions [14–16].

### Surgical technique

All operations were performed through a standard midline sternotomy with mild hypothermic cardiopulmonary bypass (CPB). In patients requiring concomitant repair of the proximal aortic arch, an open distal anastomosis was performed during a brief period of deep hypothermic circulatory arrest with retrograde cerebral perfusion, or under moderate hypothermia with selective antegrade cerebral perfusion. Aortic root replacement was performed according to the modified Bentall-de Bono operation [2]. Our technique for the replacement of the ascending aorta with aortic root remodelling (ARR) without coronary artery reimplantation has been described previously [13].

**Fig. 1:**
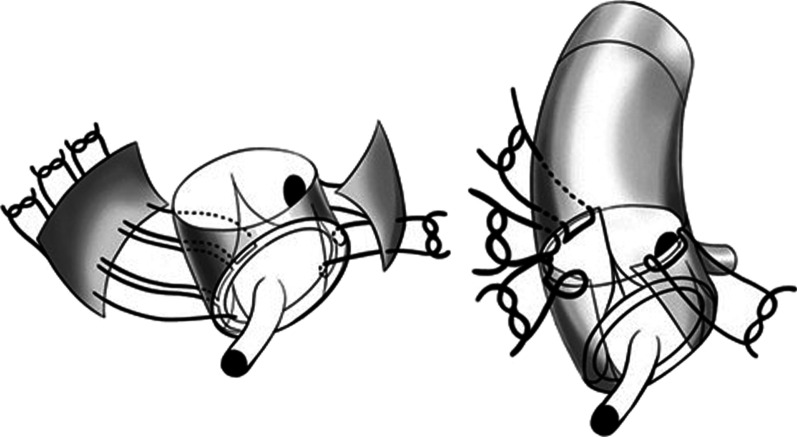
Aortic root remodelling is performed by anchoring external rectangular and square pieces of Dacron graft to the sewing ring of the prosthetic aortic valve along the non-coronary sinus region, and junction of the left and right coronary sinus region, respectively, of the replaced aortic root

Specifically for the ARR procedure, almost all aortic root tissue is excised, except for the coronary ostia, with the proximal limit of the ascending aorta resection extending 2 cm distally from the coronary ostia. The aortic valve is then replaced using 2–0 polyester interrupted sutures with pledgets placed on the ventricular surface of the aortic annulus. Three 4–0 polypropylene sutures are placed horizontally through the aortic valve sewing ring and externally through the adjacent aorta of the non-coronary sinus along with an external Teflon strip, and tied but not divided. A separate interrupted 4–0 polypropylene suture is placed through the aortic valve sewing ring externally through the adjacent aorta at the junction between the left and right coronary cusps, and tied but not divided. The ascending aorta is then replaced with a Dacron interposition tube graft with completion of the distal aortic anastomosis first. The heart and interposition tube graft are de-aired. Two pieces of Dacron graft are then prepared, a larger rectangular and a smaller square piece, to cover the aortic root regions corresponding to the previous non-coronary sinus, and the junction between the previous left and right coronary sinuses, respectively. The three previously-placed polypropylene sutures are then passed through the lower long edge of the rectangular Dacron graft piece, tied, and divided (Fig. 1). Another three 4–0 polypropylene sutures are then placed between the upper long edge of the rectangular Dacron graft piece and the adjacent interposition tube graft. This provides additional reinforcement for this section of replaced aortic root. Finally, the square Dacron graft piece is secured along the replaced aortic root corresponding to the junction of the previous left and right coronary sinuses thereby enhancing its integrity, using the previously-placed 4–0 polypropylene suture along the lower edge, and an additional 4–0 polypropylene suture placed along the upper edge (Fig. 1).

### Statistical analysis

Continuous variables are reported as mean and standard deviation, and categorical variables as counts and percentages. Risk estimates are reported as hazard ratio (HR) with their 95% confidence interval (CI). Difference in the long-term survival between the study groups was evaluated using the Kaplan–Meier method with the log-rank test. Cox proportional hazard test was used for multivariable analysis of long-term survival. Regarding survival analysis for the treatment methods, the proportional hazard assumption was confirmed evaluating the survival probability curves and using the test based on Schoenfeld residuals (not adjusted *p* = 0.09; adjusted *p* = 0.142). Analysis of repeat surgery on the aortic root/aortic valve was performed using the Fine-Gray’s method considering all-cause death as a competing event. *P* < 0.05 was considered statistically significant for all tests. Statistical analyses were performed using SPSS v. 27.0 (IBM Corporation, New York, USA) and Stata v. 15.1 (StataCorp LLC, Texas, USA) statistical software.

## Results

A total of 181 patients underwent either a modified Bentall-de Bono procedure (104 patients) or ARR technique (77 patients). The baseline characteristics and operative data of these patients are summarized in Table 1. Patients who underwent ARR were significantly older (64.2 ± 10.7 vs. 56.8 ± 15.4 years, *p* = 0.002) and less frequently had history of prior aortic surgery than patients who underwent a modified Bentall-de Bono procedure (0 vs. 7.7%, *p* = 0.022). Otherwise, baseline risk factors were well balanced between the study groups. ARR procedures were performed with a significantly shorter aortic cross-clamp time (106 ± 38 vs. 141 ± 33 min, *p* < 0.0001) than modified Bentall-de Bono procedures. CPB durations were nevertheless comparable between the study groups (Table 1). ARR procedures were associated with numerically lower risk of 30-day mortality (1.3 vs. 4.8%, *p* = 0.192) compared to modified Bentall-de Bono procedures, but the difference did not reach statistical significance. The risk of other early adverse events was comparable between the study groups (Table 2).

**Table 1:** Baseline characteristics and operative data of the study groups

Characteristics	AVR and aortic remodelling n = 77	Modified Bentall-de Bono n = 104	*P*-value
*Clinical variables*			
Age, years	64.2 ± 10.7	56.8 ± 15.4	0.002
Age ≥ 75 years	16 (20.8)	12 (11.5)	0.089
Female	23 (29.9)	23 (22.1)	0.236
Body mass index, kg/m^2^	28.9 ± 4.9	28.2 ± 6.1	0.163
Serum creatinine, μmol/L	89 ± 64	86 ± 20	0.253
Prior myocardial infarction	3 (3.9)	5 (4.8)	1.000
Prior PCI	9 (11.7)	6 (5.8)	0.153
Prior cardiac surgery	8 (10.4)	19 (18.3)	0.141
Prior aortic surgery	0 (0)	8 (7.7)	0.022
Diabetes	4 (5.2)	4 (3.8)	0.663
Smoking habit	36 (46.8)	50 (48.1)	0.860
Hypertension	58 (75.3)	73 (70.2)	0.445
Dialysis	1 (1.3)	1 (1.0)	1.000
Pulmonary disease	8 (10.4)	17 (16.3)	0.251
Cerebrovascular disease	6 (7.8)	5 (4.8)	0.406
Peripheral arterial disease	6 (7.8)	6 (5.8)	0.589
LVEF ≤ 50%	23 (29.9)	33 (31.7)	0.789
Pulmonary hypertension	4 (5.2)	6 (5.8)	1.000
Aorta diameter at pre-operative imaging			
Sinus of Valsalva, mm	44.1 ± 8.0	47.4 ± 8.3	0.009
Sino-tubular junction, mm	43.0 ± 8.9	46.5 ± 9.9	0.021
Mid-ascending aorta, mm	47.7 ± 8.8	45.0 ± 9.0	0.098
*Operative data*			
Bicuspid aortic valve	37 (50.0)	43 (49.4)	0.942
Mechanical prosthetic valve	30 (39.5)	87 (86.1)	< 0.0001
Concomitant CABG	3 (3.9)	5 (4.8)	1.000
Hypothermic circulatory arrest	9 (11.7)	10 (9.7)	0.669
Aortic cross-clamp time	106 ± 38	141 ± 33	< 0.0001
Cardiopulmonary bypass time	182 ± 58	191 ± 65	0.268

Categorical variables are reported as counts and percentages (in parentheses). Continuous variables are reported as mean and standard deviation

*CABG* coronary artery bypass grafting, *LVEF* left ventricular ejection fraction, *PCI* percutaneous coronary intervention

**Table 2:** Early outcomes of the study groups

Outcomes	AVR and aortic remodelling n = 77	Modified Bentall-de Bono n = 104	*P*-value
In-hospital mortality	1 (1.3)	4 (3.8)	0.396
30-day mortality	1 (1.3)	5 (4.8)	0.192
Intra-aortic balloon pump	3 (4.1)	7 (6.9)	0.524
Early re-operation	7 (9.1)	19 (18.3)	0.082
Re-operation for bleeding	6 (7.8)	4 (3.8)	0.328
Re-operation for cardiac complications	0	13 (12.5)	0.001
Re-operation for sternal wound complications	1 (1.3)	6 (5.8)	0.241
Deep sternal wound infection	2 (2.6)	1 (1.0)	0.394
Cerebrovascular complications			0.220
Stroke	0	2 (1.9)	
Transient ischemic attack	0	2 (1.9)	
Dialysis	1 (1.3)	2 (1.9)	1.000
Hospital stay, days	10.8 ± 5.6	11.6 ± 7.4	0.616

Categorical variables are reported as counts and percentages (in parentheses). Continuous variables are reported as mean and standard deviation

During the 7-year follow-up, 4 patients (3.8%) underwent repeat surgery on the aorta (3 on the aortic root/aortic valve) after modified Bentall-de Bono procedure: one patient underwent aortic arch and descending aortic aneurysm repair, one patient underwent surgery for aortic prosthetic valve endocarditis, one patient underwent repair of a pseudoaneurysm of the right coronary button and one patient underwent repair of a pseudoaneurysm originating from the aortic annulus. No patients who underwent ARR required re-operation during the follow-up period.

**Fig. 2:**
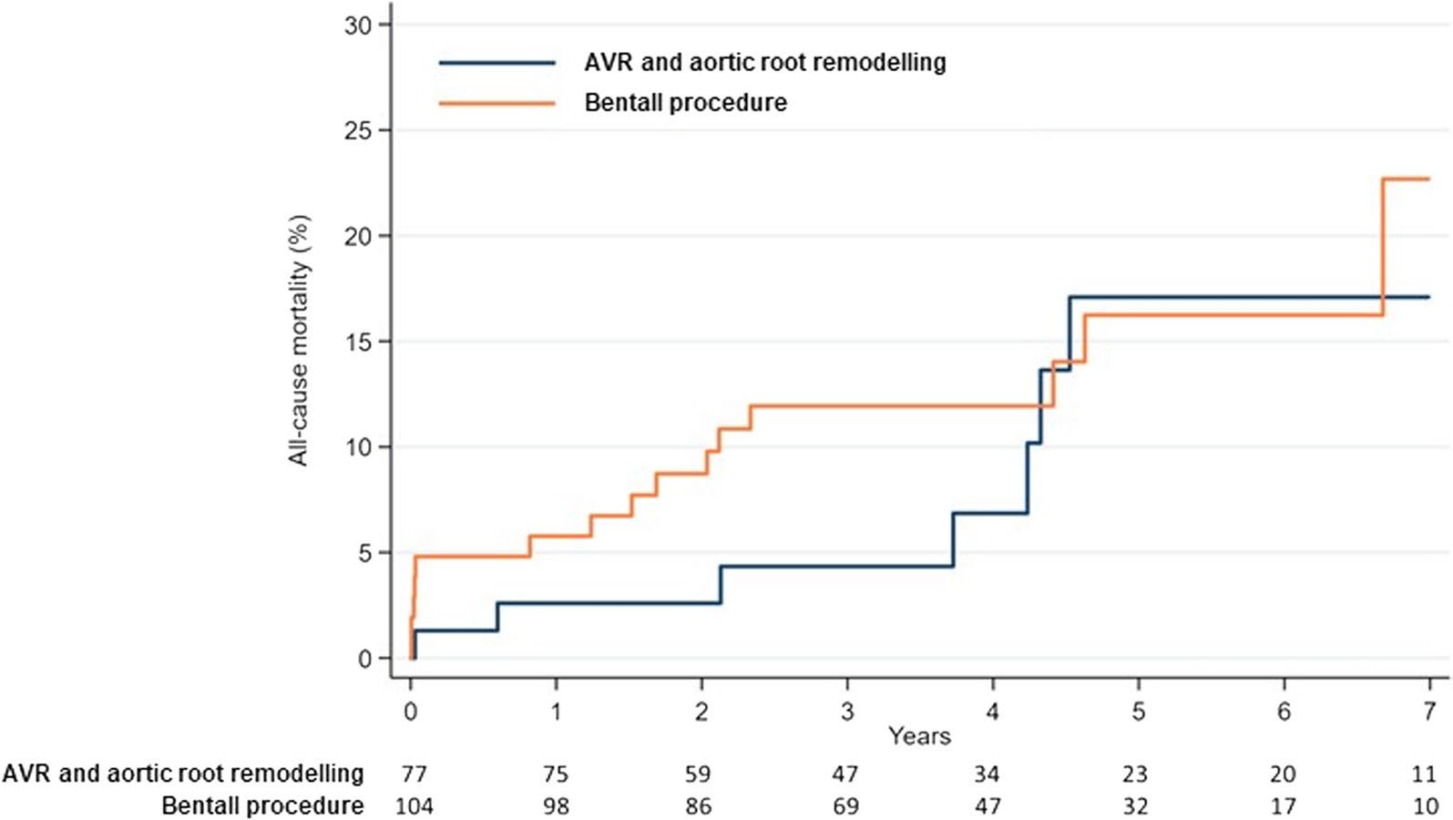
Kaplan–Meier estimates of late survival between patients undergoing aortic valve replacement with aortic root remodelling and modified Bentall-de Bono procedure

**Fig. 3:**
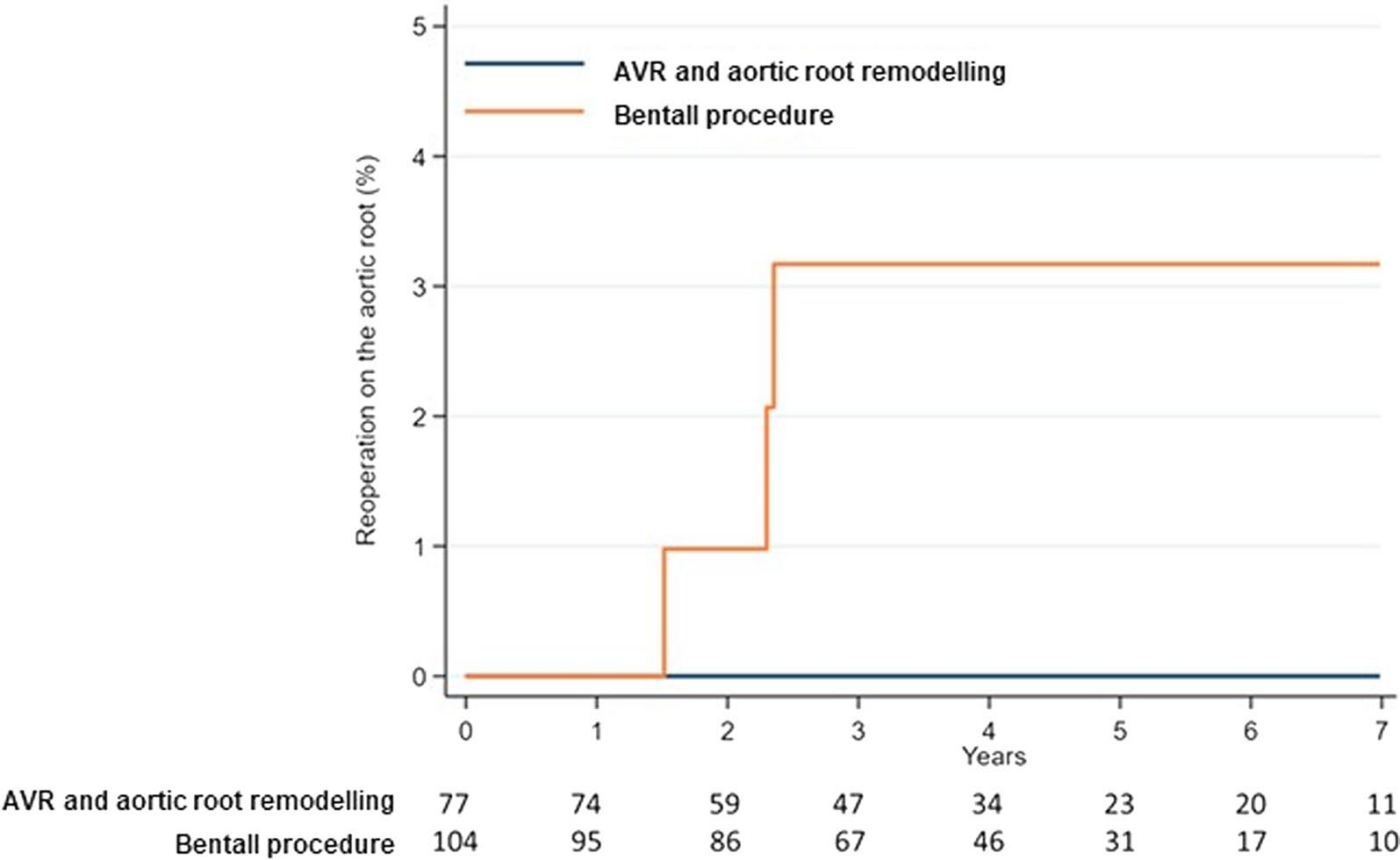
Competing risk estimates of repeat operation on the aortic root/aortic valve in patients undergoing aortic valve replacement with aortic root remodelling and modified Bentall-de Bono procedure

At 7-year follow-up, mortality rate was 17.1% after ARR and 22.7% after a modified Bentall-de Bono procedure (Log-rank test, *p* = 0.519) as shown in Fig. 2. The cumulative incidence of repeat procedure on the aortic root/aortic valve was 3.2% after a modified Bentall-de Bono procedure and 0% after ARR (Fine-Gray’s test p-value not provided) as shown in Fig. 3. Cox proportional hazard test showed that, when adjusted for other independent risk factors (age, HR 1.08, 95% CI 1.03–1.13; pulmonary disease, HR 3.59, 95% CI 1.38–9.33; prior cardiac surgery, HR 4.27, 95% CI 1.53–11.88), the treatment method did not have any impact on late mortality (adjusted HR 1.411, 0.58–3.44). Competing risk adjusted for age showed that the modified Bentall-de Bono procedure was associated with an increased risk of repeat operation on the aortic root/aortic valve (Fine-Gray’s test, *p* < 0.0001).

## Discussion

Untreated aortic root dilatation may culminate in life-threatening acute aortic dissection or rupture [17]. To reduce the risk of these adverse events before dangerous size thresholds are exceeded, current guidelines recommend elective replacement of the aortic root and/or ascending aorta at a diameter of 5.5 cm, or 4.5 cm with aortic valve dysfunction, in the absence of additional risk factors or aortopathy [1].

The standard surgical management of aortic root aneurysms irrespective of aortic valve function is the modified Bentall-de Bono procedure, whereby the entire aortic root complex including the aortic valve is replaced with a composite valve-graft alongside coronary ostial reimplantation [18]. Whilst yielding excellent short- and long-term outcomes [19–21], modified Bentall-de Bono aortic root replacement is associated with prosthetic valve-related thromboembolism and endocarditis, and bleeding from the suture line of the reimplanted coronary buttons. To maintain the integrity of the native aortic valve and sinuses, a valve-sparing root replacement procedure was pioneered by Yacoub in 1983 as a root remodelling technique [22], which may however risk progressive annular dilatation and aortic valve incompetence owing to a lack of annular stabilisation [23]. To address this issue, David and colleagues proposed a reimplantation technique in 1992 [24] offering robust aortic root support and enhanced haemostasis.

Despite these refinements in surgical technique and the availability of dedicated aortic grafts, the perioperative risks of aortic root replacement are not negligible, and may not be well-tolerated in high-risk populations presenting with advanced age, left ventricular dysfunction and multiple systemic comorbidities, and those undergoing emergency or redo operations [25, 26]. In these patient groups, a reduction in cardiopulmonary bypass and myocardial ischaemia times may be beneficial to mitigate the surgical insult and improve outcomes. Furthermore, manipulation and re-anastomosis of fragile or calcified coronary ostia may be hazardous, and in combination with residual diseased aortic wall around the reimplanted coronary buttons, may risk bleeding, coronary ostial torsion or stenosis, and late pseudoaneurysm formation. Such technically-demanding aortic procedures may be difficult to reproduce, for example when undertaken out-of-hours by less-experienced surgeons performing emergent aortic surgery.

**Fig. 4:**
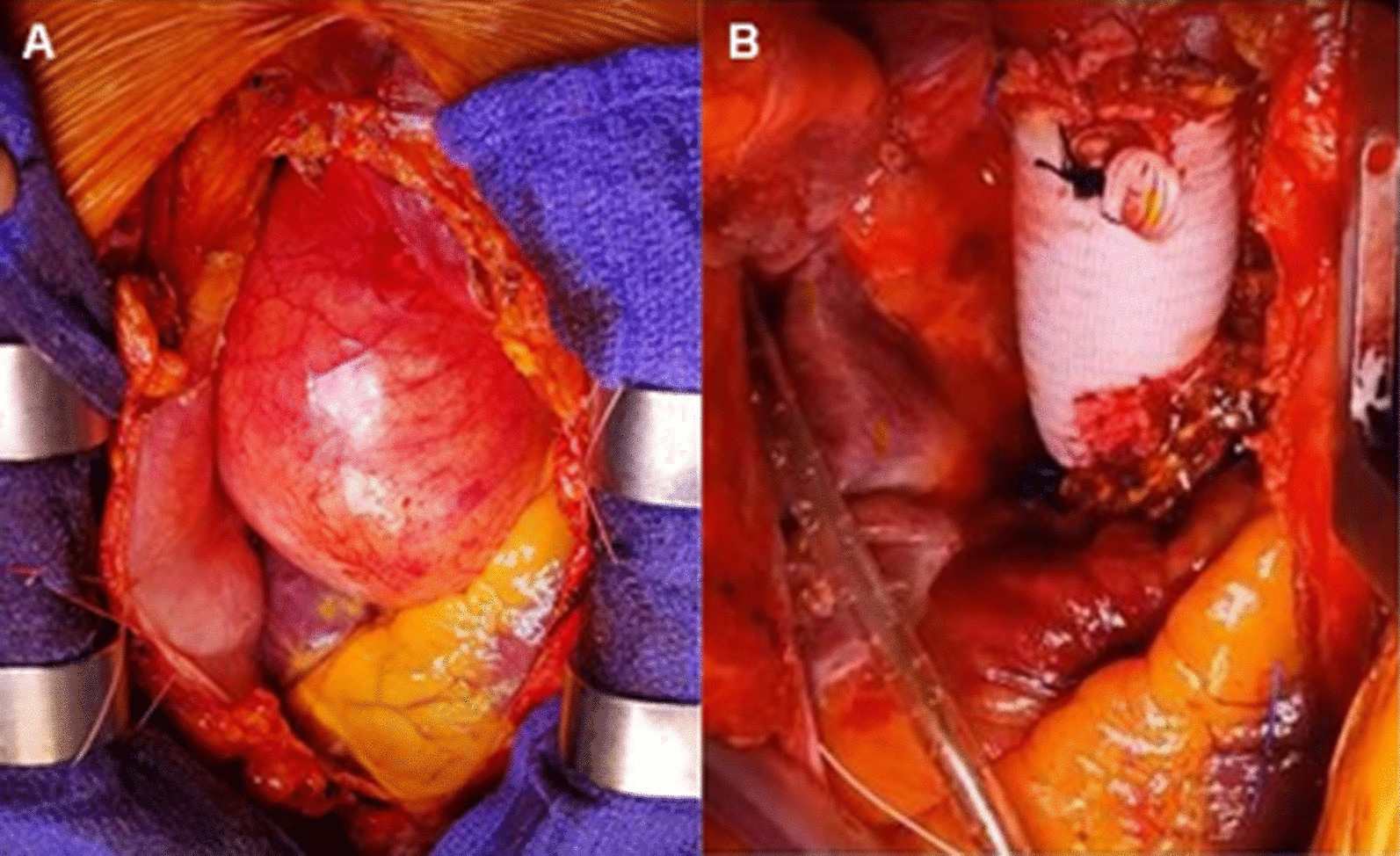
Intra-operative photographs showing an aneurysm of the aortic root and ascending aorta before ARR (**A**) and following ARR (**B**)

Since 2013 at our centre, we have routinely employed a modified remodeling technique [13] for aortic root replacement without coronary ostial mobilization or reimplantation, with aortic valve replacement when the aortic valve is not amenable to repair (Fig. 4). Our standardized ARR technique is easily reproducible without demanding more advanced technical skills in aortic surgery, and can therefore be accomplished in less time and with greater ease than a conventional modified Bentall-de Bono procedure. It involves just two aortic anastomoses, without the additional two required for coronary button reimplantation in a modified Bentall-de Bono procedure. Our approach is therefore particularly appealing when mentoring early career surgeons and training residents, although all cases in the present series were performed by an experienced aortic surgeon, and could be a viable strategy for emergency and more complex redo surgery. In the present series however, we excluded emergency operations, since the varying distribution and severity of aortic involvement may preclude a consistent operative approach. Our ARR technique incorporates aortic valve replacement and thus affords excellent aortic annular stabilization. We have not extended this technique to valve-sparing root replacement currently. The ascending aorta is excised to the level of the sino-tubular junction, leaving just a small rim of native aortic root wall. By circumferentially “wrapping” the remaining sub-coronary plane of the aortic root with an external Dacron strip, this non-resected region of the native aorta is double-reinforced, with inner Teflon felt and outer Dacron, to mitigate the potential risk of late dilation. Another important characteristic of our ARR technique is the placement of five interrupted Ethibond sutures at the midpoint of the posterior wall of both proximal and distal aorta-graft anastomoses. In our previous experience of combining this with the apposition of a 2 mm Teflon strip along the outer edge of the aorta, very secure haemostasis along the anastomotic lines is achieved.

We compared the intra-operative, early and longer-term outcomes of 77 patients undergoing our ARR technique with 104 undergoing a modified Bentall-de Bono procedure. The more straightforward nature of the ARR technique is reflected in the significantly shorter duration of aortic cross-clamping with ARR than with modified Bentall-de Bono procedures (106 ± 38 vs. 141 ± 33 min, *p* < 0.0001), although CPB durations were comparable between treatments (182 ± 58 vs. 191 ± 65 min, *p* = 0.268). In-hospital and 30-day mortality was also numerically lower in those undergoing ARR. Whilst no cerebrovascular complications occurred with ARR, four events occurred following modified Bentall-de Bono procedures, although it is difficult to attribute these directly to the surgical technique utilised. Proximal aortic replacement significantly alters normal haemodynamics within the aortic root and promotes platelet activation through a variety of mechanisms [25], including non-physiological flow patterns arising following mechanical valve implantation, exposure to the vascular graft and associated conditions of low shear stress, and possible micro-emboli originating from coronary ostial anastomoses [26]. Thus the reduced incidence of cerebrovascular events observed in patients undergoing ARR could be partially attributed to the avoidance of coronary ostial manipulation.

Structural valve deterioration, prosthetic valve endocarditis and pseudoaneurysm formation represent the principal causes for late re-operation following aortic root replacement. Importantly on longer-term follow-up, there were no re-operations on the aortic root in those undergoing ARR, suggesting the durability of the ARR technique at least over the duration of follow-up. We postulate that the double-reinforcement of the non-resected aortic root in our ARR technique may sufficiently strengthen this region of the aortic root and reduce late pseudoaneurysm formation. The re-operation rate of 3.8% in patients undergoing a modified Bentall-de Bono procedure in the present series is acceptably low and matches that of others [8–12]. Indeed, in our analysis, competing risk adjusted for age demonstrated that Bentall procedure was associated with increased risk of re-operation on the aortic root/aortic valve (Fine-Gray’s test, *p* < 0.0001). After 7 years’ follow-up, mortality was numerically lower after ARR at 17.1% compared to after modified Bentall-de Bono procedure at 22.7%, although late mortality was not influenced by surgical technique and is more likely a consequence of the comorbidity burden inherent to this patient population.

This study aimed to compare early and late outcomes of an aortic root remodelling technique without coronary reimplantation with the modified Bentall-de Bono procedure. Being a retrospective observational study of consecutive patients, it is subject to selection bias. The patient sample sizes were relatively small and patients were not randomised to undergo either surgical technique. However all surgical procedures were performed by a single experienced surgeon to ensure reproducibility. The study had a relatively short duration of follow-up of 7 years, meaning that some complications which would have been expected to develop could not have been detected. Certainly, extended follow-up is necessary to determine the late implications of leaving behind even a small amount of residual native aortic root tissue in this ARR technique.

In conclusion, we present a novel aortic root remodelling technique without coronary ostial reimplantation, affording secure annular stabilisation and external reinforcement of aortic root. It offers reduced myocardial ischaemia time, and lower post-operative mortality and re-operation rates compared to a modified Bentall-de Bono procedure. The ARR technique represents a safe, feasible and durable adjunct in the surgical management of combined aortic root and ascending aortic disease.

## Data Availability

The datasets used and/or analysed during the current study are available from the corresponding author on reasonable request.

## Abbreviations

ARR: Aortic root remodelling
CPB: Cardiopulmonary bypass
CI: Confidence interval
CT: Computed tomography
HR: Hazard ratio
NICOR: National Institute for Cardiovascular Outcomes Research
VARC-2: Valve Academic Research Consortium-2
